# Feasibility study of a digitalized nurse practitioner-led intervention to improve medication adherence in type 2 diabetes patients in Dutch primary care

**DOI:** 10.1186/s40814-021-00892-2

**Published:** 2021-08-07

**Authors:** Stijn Hogervorst, Marcel Adriaanse, Hella Brandt, Marcia Vervloet, Liset van Dijk, Jacqueline Hugtenburg

**Affiliations:** 1 Department of Health Sciences, Faculty of Science, Vrije Universiteit, Amsterdam, The Netherlands; 2 Amsterdam Public Health Research Institute, Amsterdam UMC, Location VUMC, De Boelelaan 1117, 1081 HV Amsterdam, The Netherlands; 3 Department of Pharmaceutical Care, Nivel, Netherlands Institute for Health Services Research, Utrecht, The Netherlands; 4 Department of PharmacoTherapy, Epidemiology & Economics (PTEE), Faculty of Mathematics and Natural Sciences, Groningen Research Institute of Pharmacy, University of Groningen, Groningen, Netherlands; 5 Department of Clinical Pharmacology and Pharmacy, Amsterdam UMC, Location VUMC, De Boelelaan 1117, 1081 HV Amsterdam, The Netherlands

**Keywords:** Medication adherence, Type 2 diabetes mellitus, Implementation, Feasibility study, Primary care, Collaboration, Community pharmacists, Nurse practitioner

## Abstract

**Purpose:**

The purpose of this feasibility study was to improve and implement an intervention aimed at enhancing medication adherence in sub-optimally controlled and non-adherent type 2 diabetes (T2DM) patients in primary care.

**Methods:**

Four phases were completed: (1) context analysis, (2) collaboration protocol development, (3) digitalization, and (4) process evaluation. Two community pharmacies and seven general practices participated. In phase 1, two focus groups were conducted, of which one with healthcare providers (HCP, *N* = 5) and one with patients (*N* = 11). In phase 4, four semi-structured interviews and one focus group (*N* = 6) were conducted with healthcare providers. The goal of these focus groups and interviews was to obtain insights into current care to support medication adherence (phase 1), opportunities for collaboration (phase 2) and process evaluation (phase 4). Data were analyzed in Atlas.ti using thematic analyses.

**Results:**

Both T2DM patients and HCPs considered medication adherence vital. Suboptimal collaboration between HCPs and unreliable ways to monitor medication non-adherence appeared important barriers for adequate care to support medication adherence (phase 1). The nurse practitioner (NP) was chosen as the interventionist with supportive roles for other HCPs (phase 2). All components of the intervention were digitalized (phase 3). The implementation of the digitalized intervention was reported to be suboptimal (phase 4). Main reasons were that pharmacy refill data were unreliable, NPs experienced difficulties addressing medication non-adherence adequately and collaboration between HCPs was suboptimal.

**Conclusions:**

The medication adherence enhancing intervention was successfully digitalized, but implementation of the digitalized intervention appeared not feasible as of yet.

**Supplementary Information:**

The online version contains supplementary material available at 10.1186/s40814-021-00892-2.

## Contributions to the literature

- Medication adherence interventions appear difficult to implement into routine primary care. This paper describes the implementation of a pragmatic medication adherence intervention in detail.

- Input from involved healthcare providers has shown that there are important prerequisites for successful implementation of medication adherence interventions in primary care, such as better Information and Communication Technology (ICT) systems to monitor and select non-adherent patients and a better collaboration between general practitioners (GPs) and pharmacies.

- Medication non-adherence proved a challenging topic for nurse practitioners to address. Future studies should focus on exploring ways to make difficult topics such as medication non-adherence easier to talk about with patients for nurse practitioners.

## Introduction

Type 2 diabetes mellitus (T2DM) is a highly prevalent chronic condition responsible for high morbidity and mortality rates globally [1, 2]. A cornerstone of T2DM treatment is optimal glycemic control. This can be achieved by following lifestyle recommendations and taking glucose lowering medication as prescribed [3, 4]. However, only 50% of people with T2DM have optimal glycemic control (A1c ≤ 53 mmol/mol) [5]. Not reaching these target levels increases symptom severity, diabetes-related hospitalization, and mortality [3, 4, 6] and is often attributed to patients not taking the medication as agreed upon with their clinician [7].

To support people with T2DM with taking their medications, several interventions have been developed. However, in a review including 52 studies only 9 interventions managed to improve both A1c and medication adherence levels [8]. Common success factors of these nine interventions include focusing solely on non-adherent sub-populations and tailoring the intervention to the perceived barrier(s) by the patient [9, 10]. Mainly due to their complex nature, interventions that share these common success factors appeared difficult to implement in clinical practice [11, 12]. These interventions are complex because adequate medication adherence support in T2DM patients often requires continuous monitoring of the patient and close collaboration between different healthcare providers [13].

In recent years, T2DM care has shifted from hospital care towards primary care in the Netherlands. As a result, currently, 62% of Dutch general practitioners (GPs) work with at least one nurse practitioner (NP) who is responsible for T2DM care [14]. Thus, the urge to improve medication adherence of T2DM patients in primary care has increased. This shift in care asks for the implementation of interventions aimed at improving medication adherence of T2DM patients in primary care, where collaboration between community pharmacy (CP) teams and GP teams can be facilitated.

The current feasibility study builds upon a previous study by Adhien et al. [15], an intervention developed for CP teams in the Netherlands aimed to improve medication adherence in T2DM patients (Table 1). The ‘Support for Diabetes’ intervention proved to be engaging and feasible to implement in community pharmacies, but suboptimal co-operation between pharmacists and GPs and lack of time were obstructions for implementation on a wider scale.

**Table 1:**
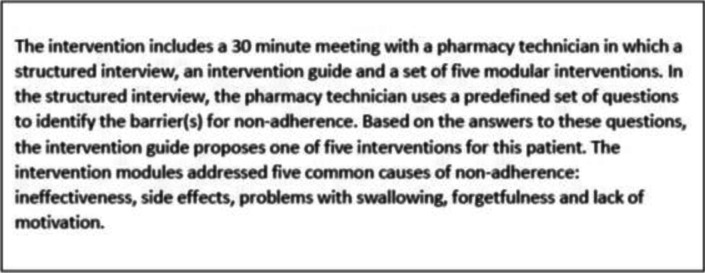
Short description of the ‘Support by for Diabetes’ 1 intervention by Adhien et al. [15]

Previous research in The Netherlands showed that collaboration between GP and CP teams is often subpar [16]. Collaboration is an important prerequisite to implement successful medication adherence interventions [15], because medication care of T2DM patients relies on a strict division of most of the tasks but not of all tasks. In the Netherlands, GPs are responsible for diagnosis of T2DM. The nurse practitioners (NPs) are responsible for the daily care for T2DM patients [17]. Medication is provided by pharmacies. Tasks of care to support medication adherence, such as provision of information and inquiring about medication adherence, are less clearly divided. Fine tuning of care to support medication adherence is therefore necessary, as an unclear division of tasks might lead to conflicting information for patients or tasks not being carried out at all. The current study tried to tackle this barrier by clarifying the division of tasks by developing a collaboration protocol based on HCPs needs and wishes.

Another barrier found by the study by Adhien et al. was a perceived lack of time [15]. Digitalizing different tasks is a way of limiting the human effort needed to carry out a task, for example by automatically gathering data on patient refill adherence [18]. Digitalization in the healthcare sector usually involves information and communication technology (ICT) applications and has proven to be effective in decreasing time spent on administrative tasks and increasing time spent on direct care previously [19]. The current study digitalized the intervention and all of its components in order to decrease the time spent on administrative tasks and therefore lower the overall time spent on the intervention.

Designing effective collaboration and workable ICT applications for HCPs is challenging as well, because a good ‘fit’ between the program and the context is critical [11]. This means taking in to account the uniqueness of organizations, the existing collaboration between HCPs, the existing division of tasks, and the wishes and needs of stakeholders [12]. Gaining knowledge on these factors requires a detailed understanding of the specific context in which an intervention is implemented and perspectives of different actors involved in the intervention.

This study aimed to improve the ‘Support for Diabetes’ intervention by Adhien et al. in three ways: (i) a collaboration protocol was developed, (ii) the intervention was digitalized, and (iii) both CP and GP teams were involved in conducting the intervention, with the NP as the interventionist. This feasibility study aimed to improve and implement an intervention enhancing medication adherence in sub-optimally controlled and non-adherent type 2 diabetes (T2DM) patients in primary care. Qualitative research methods were used to gain insights into needs and wishes of healthcare providers (HCPs) and patients as well as to evaluate the project.

## Methods

### Study design

In this feasibility study, an adapted version of the ‘Support for Diabetes’ intervention aimed at improving medication adherence in non-adherent and sub-optimally controlled (A1c of < 7% (53 mmol/mol)) T2DM patients was developed, implemented, and evaluated in Dutch primary care. The intervention was improved upon by digitalizing all of its components and by stimulating a productive collaboration between GP and CP teams by developing a protocol for collaboration based on input from patients and HCPs. A schematic overview of the improved and digitalized ‘Support for Diabetes’ intervention can be found in Additional file 1: Appendix F. This feasibility study consists of four phases: (1) context analysis, (2) collaboration protocol development, (3) digitalization, and (4) implementation of the intervention.

### Recruitment of primary care healthcare providers

As this project uses a digitalized intervention embedded in the Dutch routine care registration systems Pharmacom (CP) and Medicom (GP), only CP/GP teams that use these systems were eligible for participation in this project. Eligible GP/CP teams were identified by the developers of the registration systems. They were selected and approached by e-mail by SH in which their interest in participation in the project was requested. Two healthcare centers and their eligible T2DM patients participated in this project. Involved HCPs were GPs, NPs, CPs, and pharmacy assistants. Healthcare centers are primary care centers in which generally a single community pharmacy and multiple GPs are located. One of the healthcare centers was located in the Dutch city of Amstelveen, and the other in the Dutch city of Katwijk.*’*

### Phase 1–context analysis

In the first phase, two focus groups among both patients and HCPs were held to gain insight into the context in which the implementation would be tested. The goal was to gain insights into the current standards of care to support medication adherence.

### Phase 2—collaboration protocol development

The results from the focus group with healthcare providers and patients were used to develop a collaboration protocol for all HCPs in the intervention (see Additional file 1: Appendix D). This collaboration protocol ensures that both CP and GP teams are involved in the project and enables a clear division of tasks. Additionally, a 2-h training for all HCPs was developed, which was offered on two separate occasions for both healthcare centers by SH and JH. The training focused on theory about medication adherence, such as the different phases of medication (non-)adherence, determinants, and ways to address medication non-adherence [9, 20]. The training also included an explanation of the collaboration protocol and division of tasks as well as practicing with new ICT protocols by using them on test patients.

### Phase 3—digitalization

The original intervention by Adhien et al. was used by HCPs as printouts, whereas the current intervention is fully digitalized [15]. Digitalization of the intervention and the collaboration protocol was done in an existing and linked GP and CP ICT system (Pharmacom/Medicom). See Additional file 1: Appendix E for an example of the digitalized intervention. Pharmacom holds the medication history among others and is accessible by pharmacists. Medicom is accessible by GPs and holds both the medication history and the patient records. Pharmacom is the largest ICT system for CPs in the Netherlands and Medicom is among the largest for GPs. This ensured that the intervention was embedded in a familiar ICT environment accessible to all HCPs. In addition, collaboration was further stimulated by ICT as the filled out intervention was digitally and automatically shared between the CP and GP teams.

### Phase 4—process evaluation

The improved and digitalized intervention ‘Support for Diabetes’ was implemented in two healthcare centers in the Netherlands. ‘T2DM patients were eligible for the intervention when they were both non-adherent to oral antidiabetics based on pharmacy refill data (< 80% of days covered was considered non-adherent) and sub-optimally controlled (A1c of < 7% (53 mmol/mol)). Due to the focus on feasibility, patients’ outcomes are not reported in this paper. For a description of the improved and digitalized ‘support for diabetes’ intervention, see Additional file 1: Appendices D (collaboration protocol) and F (schematic overview of the intervention).

A process evaluation was conducted using qualitative research methods, to gain insights into the implementation of the improved and digitalized ‘Support for Diabetes’ intervention. Quantitative data was collected from participating patients from the routine checkups available in GP and CP ICT systems to see how convenient it was to retrieve those data.

### Data collection

**Fig 1:**
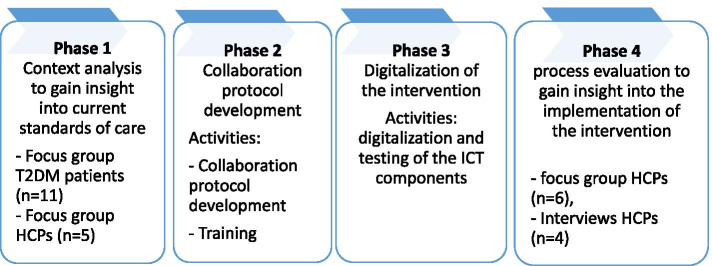
Overview of the different phases and measurements in the ‘Support for Diabetes’ project.

Data was collected in phase 1 and phase 4 (Fig. 1). In phase 1, two focus groups were held by SH, MA, JH, and HB. Purposive sampling was used to select HCPs with varying functions for the first focus group (*n* = 5) from one of the participating primary care healthcare centers. HCPs time was compensated for by the project group according to the standard hourly wages of the staff. Patients for the second focus group (*n* = 11) in phase 1 were randomly selected by checking prescriptions and selecting all patients using T2DM medication (i.e., Metformin or Gliclazide). Patients were selected by one pharmacist from the pharmacy database and invited by telephone to participate in the focus group.. All T2DM patients who participated in the focus group received their medication from the same pharmacy, but attended different GPs. T2DM patients received a gift card for participation.

Separate topic lists for patients and HCPs can be found in Additional file 1: Appendices A and B. Topics for the focus group with T2DM patients were ‘medication adherence,’ ‘medication support and information provided,’ and ‘multidisciplinary care and collaboration.’ Topics for the focus group with HCPs were medication adherence, communication, collaboration, and satisfaction with current standards of care to support medication adherence.

In phase 4, a process evaluation was held to gain insight into the implementation of the intervention. This consisted of four interviews (two GPs and two NPs from the Katwijk location) and one focus group with HCPs who participated in the intervention (*N* = 6; two GPs, one CP, and three NPs from the Amstelveen location) were held by SH, MA, and HB. The topic list was based on the RE-AIM framework [21]. The five RE-AIM domains (Reach, Effectiveness, Adoption Implementation, Maintenance) were used as topics. The complete topic list can be found in Additional file 1: Appendix C. The RE-AIM framework aims to increase consistency in research findings and to help translate research into practice.

All focus groups and interviews were audio recorded with consent and transcribed verbatim. With the exception of one interview by phone, all interviews and focus group were held at the healthcare center that patients and HCPs were affiliated with. Focus groups lasted about 2 h on average, interviews about 1 h.

### Data analysis

Thematic analysis was used to analyze qualitative data [22]. This approach emphasizes identifying, analyzing, and interpreting patterns of meaning (i.e., themes) within the data.

Different investigators were involved in the analysis process. To increase the credibility of this study two researchers (SH and HB) coded each interview transcript independently and verified each other’s work. All emerging themes in the transcripts were marked, labeled, and compared between the two researchers to increase dependability and to reach consensus [23]. The themes of all transcripts were grouped together to create a list of recurring themes. Atlas TI 5.1.7. software was used for coding and structuring of themes (Table 2).

**Table 2:**
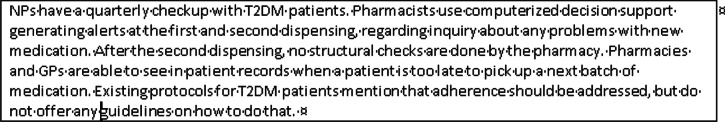
Current standard of care to support medication adherence care for T2DM patients

## Results

### Participants

Table 3 provides an overview of all participants in this study. In phase 1, the focus group of T2DM patients (*N* = 11) had participants with ages ranging from 43 to 84 (average age = 69). The focus group consisted of 7 females and 5 males. The focus group with HCPs (*N* = 5) in phase 1 consisted of two GPs, a NP and two pharmacy assistants from the Amstelveen location. The focus group in phase 4 (*N* = 6) consisted of two GPs, one CP, and three NPs from the Amstelveen location. Additionally, four interviews were held with HCPs from the Katwijk location, of which 2 interviews with GPs and two interviews with NPs.

**Table 3:** Characteristics of patient and HCPs that participated in the focus groups during phases 1 (context analysis) and 4 (testing the implementation)

**Patients (phase 1, *N* = 11)**		
-Gender (*n*)	Female	7
	Male	4
-Age	Range	43–84
	Mean	69
-Chronic diseases (*n*)	T2DM	11
	Hypertension	8
	High cholesterol	9
**Healthcare providers** (*N* = 15)		
*Phase 1 (n* = *5)*		
-Gender	Female	4
	Male	1
-Function	General practitioner	2
	Nurse practitioner	1
	Pharmacist	1
	Pharmacy technician	1
*Phase 4 (n* = *10)*		
-Gender	Female	6
	Male	4
-Function	General practitioner	3
	Nurse practitioner	5
	Pharmacist	2

### Phase 1—context analysis

Findings are organized around three main themes found through thematic analysis (see Table 4). These themes are ‘perspectives of care to support medication adherence by patients and HCPs,’ ‘current standards of care,’ and ‘barriers in care to support medication adherence’ Quotations are used to illustrate the themes and are presented in Table 5.

**Table 4:** Overview of themes and main results in phase 1–context analysis to gain insights into the current standards of care to support medication adherence for people with T2DM

Theme	Focus group T2DM patients (*N* = 11)	Focus group healthcare providers (*N* = 5)
Perspectives of care to support medication adherence by patients and HCPs	Adequate medication adherence is vital for T2DM patients	Medication adherence is seen as the responsibility of the patient. HCPs solely inquire about medication adherence when blood levels are elevated, often with a single question
Current standards of care	Suboptimal communication and collaboration between HCPs causes patients to feel like they have to be critical and attentive to the care they receive	GP and CP staff members have different work ethics and work flow that do not align, causing communication and collaboration to be suboptimal
Barriers in care to support medication adherence	Not applicable	HCPs have no reliable way to electronically monitor patients’ medication adherence, which results in non-adherence often not being addressed

**Table 5:** Quotes for HCPs and patients in phase 1—context analysis to gain insights into the current standards of care to support medication adherence for people with T2DM, based on a focus group with patients *N* = 11 and a focus group with HCPs *N* = 5

Quote	Description	By whom
1.1 Perspectives on care to support medication adherence by healthcare providers and T2DM patients		
**1.1.1**	When I am guarding the door at night, because I work as a porter, that is a time when I use my Simvastatin. I am supposed to take those before going to bed, but those nights are different and I had to figure out how to deal with that by myself	Patient 1
**1.1.2**	Pharmacists are only in it for the money. The more pills they can give out, the more they earn	Patient 2
**1.1.3**	Well, sometimes there are patients at the front desk who complain about the size or the taste of their medication. Especially calcium chew, people don’t like that one. Or people might complain about the brand of the medication. But other than that, we don’t hear many problems, but I also think that there are not that many patients in our pharmacy that are non-adherent	Pharmacy assistant 1
**1.1.4**	I believe adherence is the responsibility of the patient. However, there is an exception in case of medical danger. If a patients’ blood sugar is so high that they are in danger or might end up in a coma, then I believe that we should take action	Nurse practitioner 1
1.2 Current standard of care		
**1.2.1**	The NP often tells me that I can pick up the medication straight after our consult, but even when I return to the pharmacy the next day, they seem to be completely unaware of this request	Patient 3
**1.2.2**	Yes, the NP sometimes forgets to call me back, but that is understandable, because everybody at the GP is always working so hard	Patient 1
**1.2.3**	I am a GP, and therefore I am journal focused. When I look up a patient on my PC, the first thing I do is open the journal. Every HCP is different in this regard, I believe pharmacists are more medication oriented. They look up a patient and only look at their medication history. Therefore a pharmacist might add some relevant information under medications, but I might never see that because that is just not how I work	General practitioner 1
**1.2.4**	Yes, because a patient then says to me ‘I’m having these and these symptoms, and the pharmacy told me to quit my medication immediately’. Then I think to myself, I don’t believe any of that, but what am I to do? Tell the patient to wait for a second, while I phone the pharmacy and ask them ‘I have a patient here who claims you have said this and this, is that true?’. That is ridiculous of course	General practitioner 2
1.3 Barriers in the care to support medication adherence		
**1.3.1**	I also believe the repeat dispensing, that is really something that makes me lose control. Sometimes patients call me and ask me questions about medication, but I really do not know why they are taking that medication. That might be something they took years ago, but the repeat dispensing service just keeps giving that to patients without my knowledge	General practitioner 2
**1.3.2**	That step [i.e. what to do when you noticed a non-adherent patient], if that is clear, then you make it a lot easier on others to identify non-adherent patients. Because otherwise you might say ‘yes, that person is here, but what now?’. Then I might have done my part and given the signal, but the follow-up action is completely unclear	Nurse practitioner 1

#### Theme 1.1—perspectives on care to support medication adherence by T2DM patients and healthcare providers

##### Medication adherence is vital for T2DM patients

Adequate medication adherence is seen as vital by T2DM patients. Most important reasons are the prevention of hypoglycemic events (i.e., low blood sugar), death, and a responsibility towards family members to take good care of ones’ health. However, patients also indicate that more complicated medication regimens result in less adherence, as well as not having the medication available when needed, mainly due to shortages or issues with delivery by the pharmacy. Patients stress the need to find their own way in sticking to difficult medication regimens (quote 1.1.1 in Table 5).

##### Nurse practitioner is the central actor for T2DM patients

Patients consider the NP as the central actor in all care surrounding T2DM and the NP was the first they would contact when they experience any issues with medication. Patients are satisfied with their relationship with their NP. The relationship between patients and the CP, however, is mediocre at best (quote 1.1.2 in Table 5). Patients perceive the CP as being a commercial institution. Reasons given by patients for that distrust are that pharmacists are not personally engaged and give unnecessary treatment or make administrative mistakes for their own benefit. Expertise and being personally interested/engaged are seen as the most important traits of a good HCP by patients. As a result, patients only disclose technical problems with medication with CP staff members, whereas they might disclose more personal issues with their NP (quote 1.1.3 in Table 5).

##### Medication adherence is a patients’ own responsibility

HCPs see medication adherence as vital, but only see a small role for themselves when it comes to care to support medication adherence and consider it the responsibility of the patient (quote 1.1.4 in Table 5). Specifically, NPs do not actively ask patients whether they understand the medical information provided with the medication. Only when patients A1c levels are considered too high, NPs actively question and inform patients regarding medication adherence. A reason NPs hesitate to address suspicion of medication non-adherence with a patient, is that they feel it could potentially break the trust bond with the patient. NPs might offer solutions such as pill boxes or medication alarms to help patients remember to take their medication.

#### Current standard of care

##### T2DM patients experience hindrance from a lack of collaboration between HCPs

Patients perceive the collaboration between HCPs as suboptimal. Patients feel like they have to be attentive and pro-active towards the care they receive. Compartmentalization in the care sector is seen as a major cause. Patients are often suffering from a lack of collaboration in the care sector, for example when a NP promises that the medication will be available the same day, but the CP is unable to provide the medication that day (quote 1.2.1 in Table 5). CPs receive mainly all the blame from patients when it comes to issues rooted in miscommunication (quote 1.2.2 in Table 5).

##### Different work flows of CP and GP staff members

GP and CP staff members have different work flows that do not necessarily align. Examples are CP staff members who use the medication prescription to write down information about a patients’ medication non-adherence for CP staff members. This information, which is also relevant for GPs, does not reach GP staff members because they do not receive the medication prescription back from the CP. GP and CP staff members share ICT systems, but GPs use the patient records, while the CP looks at the medication history and therefore often miss the information shared by the other (quote 1.2.3 in Table 5). The result is a lack of exchange of information between the GP and the CP. HCPs from GP practices and CPs therefore often communicate by telephone (quote 1.2.4 in Table 5).

#### Barriers in care to support medication adherence

The repeat dispensing service provided by CPs for patients with chronic conditions automatically generates a new prescription after a given time period, regardless whether the patient picked up the medication or not. This affects the reliability of the automatic refill adherence monitoring for T2DM patients by CPs. As a result, HCPs undividedly agreed that medication adherence data based on pharmacy refill is not trustworthy due to repeat dispensing (quote 3.1.1 in Table 5).

Moreover, due to the lack of an adequate system to monitor medication adherence in T2DM patients and a lack of guidelines, follow-up actions on a non-adherent T2DM patient are often not performed (quote 1.3.2 in Table 5).

### Phase 2—collaboration protocol development

Information collected in phase 1 emphasize the need for a collaboration protocol and guidelines for care to support medication adherence for T2DM patients. The collaboration protocol is presented in Additional file 1: Appendix D and consists of a clear distinction of tasks categorized into the topics ‘selection of patients,’ ‘signaling and plan for action,’ ‘invite patients and baseline measurements,’ ‘conducting the ‘Support for Diabetes’ intervention,’ and ‘Follow-up’. Additionally, the NP was chosen to conduct the intervention, as is preferred by both patients and HCPs.

### Phase 3—digitalization

Digitalization of the intervention was performed in the CP and GP ICT system and ensured the use of the intervention and the exchange of information by HCPs in a familiar setting. For example, input from a patient gathered during a consult for a NP is stored in this digital environment and shared electronically with the pharmacy. An example of the digital intervention ‘Support for Diabetes’ and the information exchange function is presented in Additional file 1: Appendix E.

### Phase 4—process evaluation

Findings of the process evaluation are organized around four main themes (see Table 6) and quotations were used to illustrate the themes which are presented in Table 7. These themes are ‘training and preparation,’ ‘appreciation for the intervention,’ ‘practical barriers to implementation,’ and ‘organizational barriers to implementation.’

**Table 6:** Overview of themes and main results in phase 4—process evaluation of the implementation of the improved and digitalized ‘Support for diabetes’ intervention

Theme	Healthcare providers (focus group *N* = 6 and interviews *N* = 4)
Training and preparation	The training was well received by healthcare providers, but a written summary was missing. GPs only played a minor role and would like to have been more involved in the project
Appreciation for the intervention	The intervention was seen by NPs as a valuable addition to their nurses’ toolbox. It was deemed too extensive to apply periodically, but rather to be used when deemed necessary for certain patients
Practical barriers to implementation	Healthcare providers did not consider the time invested in this project to be in proportion to the profit in health per patient. Additionally, unreliable pharmacy refill data proved to be an important barrier for implementation in this project
Organizational barriers to implementation	NPs had a hard time addressing medication adherence in patients. There was a lack of organizational support from GPs and a high level op drop out among participating HCPs. Lastly, a good established collaboration between the GPs and CP proved to be an important prerequisite for successful implementation

**Table 7:** Quotes from HCPs and patients in phase 4—process evaluation of the implementation of the improved and digitalized ‘Support for diabetes’ intervention, based on focus group, *N* = 6 and interviews *N* = 4

Quote	Description	By whom
*4.1–Training and preparation*		
4.1.1	Well of course you have to think for yourself. But it would be nice that after not doing something for two months, there is a written summary of the steps that need to be taken for this project	Nurse practitioner 1
*4.2–Appreciation for the intervention*		
4.2.1	Well for that one man who was so unsatisfied [with the care sector], I know that he also mentioned that this was the first time that he had the opportunity to talk about these things and that is something he really appreciated. For the other man I spoke, I don’t know, it was not an unpleasant conversation. He indicated that he will take his medication from now on, but I have no way of knowing whether he actually will	Nurse practitioner 2
*4.3–Practical barriers to implementation*		
4.3.1	Well, I believed that the overhead in this project was more than the outcome	General practitioner 1
4.3.2	If you sometimes participate in home visits as a part of medication reviews, you often see patients who collected giant piles of medication at home. And you can see the same thing when someone has passed away, or when someone moves. All those people are on repeat dispensing, so they pick up their medication on time but never use it	Pharmacist 1
*4.4–Organizational barriers to implementation*		
4.4.1	We have been working together for quite some years now. By now, we know how to do things and what we can ask of each other. We know each other also outside work, so by now we hardly have to ask one another to get what we want. Therefore, the collaboration protocol is not needed as hard here as it might somewhere else	Pharmacist 1
4.4.2	There were people on the list that I had known for years, and I had given this man a medication dispenser last year, so I mean, how can he even be non-adherent?	Nurse practitioner 3
4.4.3	But I think that might be the difference between a GP and a NP. A NP has a lot of trouble asking about erectile dysfunction in patients with T2DM for example, while that is a very normal question for me	General practitioner 1
4.4.4	I see some of those patients four times a year. So I mean, if patients are truly that dissatisfied with their medication, they can mention it themselves right?	Nurse practitioner 4
4.4.5	But, all of that has to do with how you see yourself. The doctor used to be a police officer, but nowadays I see myself as a coach. I therefore don’t find it intrusive to ask about a patients medication, because if they really don’t want to take it I don’t care. My role as a coach is simply to explain them what the consequences are if they don’t	General practitioner 2
4.4.6	The main role for us is to create time. If I think something is truly important as the owner of this practice, that means I have to prioritize it. So for example if we say this is an important project, then I might leave two full hours a month free in the calenders of my NPs, so they know that that is the time they have to work on this project. I have noticed in the past that that gives more structure, because I cannot expect all my employees to do it by themselves	General practitioner 3

#### Training and preparation

The training for HCPs was well received by HCPs. Mainly NPs appreciated the content of the training and the practical approach. However, these NPs also voiced their preference for a written summary at the end of the training (quote 4.1.1 in Table 7). As the intervention was mainly focused on learning new digital protocols, some NPs requested a more practical approach with complete protocols rather than screenshots of the protocols.

#### Experience with the intervention

Both HCPs and patients considered the intervention engaging and useful (quote 4.2.1 in Table 7). NPs considered the intervention a useful addition to their nurses’ toolbox. GPs and CPs reported that they had a minor role in the performance of the interventions and would like to have been more involved.

#### Practical barriers to implementation

##### Time invested per patient is not in proportion with profit in health

HCPs consider the time invested per patient not in proportion to the profit in health, because of the following reasons. First, as this project focused only on non-adherent and sub-optimally controlled T2DM patients, which proved to be the most severe and therefore most time-consuming patients. These patients appeared to have multiple problems congregate, such as mental health issues, addiction, or not speaking either Dutch or English, which made consults with them more time-consuming. A second reason was the small scale of this intervention of only 2–3 eligible patients per GP practice. Consequently, HCPs had to participate in focus groups and training sessions for only a few patients (quote 4.3.1 in Table 7). This made involved HCPs feel like the overhead costs for this project were too high compared to the small amount of patients that participated. Thirdly, NPs needed 30 min for a consult for the ‘Support for Diabetes’ intervention, rather than the 10 min per consult they are used to. Therefore NPs questioned the cost-effectiveness to address solely medication adherence during a 30-min consult.

##### Shortcomings in the identification of eligible patients

Non-adherent T2DM patients were identified electronically based on pharmacy refill data. Subsequently, the GP and NP manually checked A1c levels of the non-adherent patients. If a patient was both non-adherent and sub-optimally controlled, they were eligible for the intervention. GPs and NPs considered this second step as labor intensive. Another hampering issue with this method was that the pharmacy refill data was often incorrect due to patients staying abroad, patients that had passed away, moved to another GP or had deviant medication regimens thereby being incorrectly labeled as non-adherent. Moreover, HCPs felt that the repeat dispensing service provided by CPs (see “Barriers in care to support medication adherence” section) created a group of ‘false adherent’ patients who solely pick up their medication in order to be registered as ‘adherent’, but never actually take the medication (quote 4.3.2 in Table 7).

### Organizational barriers to implementation

Lack of established collaboration in one primary healthcare center.

A large difference in the level of implementation and uptake was observed between the two participating healthcare centers. The Katwijk location has a longstanding history of collaboration between the GP and CP, whereas in the location Amstelveen the collaboration was subpar (quote 4.4.1 in Table 7).

#### Limited organizational support by GPs

Another barrier is the lack of organizational support by GPs. Many NPs indicate that they lack the time to carry out an extra 30-min consult. GPs could play a role by prioritizing the project and scheduling dedicated time for the NPs to work on this project (quote 4.4.6 in Table 7).

#### Personal barriers to implementation

##### NPs experience difficulties in discussing medication non-adherence with patients

NPs experience difficulties in discussing medication adherence with their patients. NPs seemed to underestimate the size of the problem, especially when their patients were already advised to use a medication reminder such as an alarm or pill box (quote 4.4.2 in Table 7). In addition, NPs report not to know how to address adherence. They prefer to have a protocol with example questions for addressing non-adherence. During the project, NPs often invited patients for the intervention by phone by stating that they are non-adherent based on pharmacy refill data. This caused patients to react in a defensive manner, in which they would claim they were adherent. As a result, NPs canceled the consult with this patient. A GP stated that NPs, in contrast to GPs, are not trained to talk about delicate topics (quote 4.4.3 in Table 7). NPs also mentioned that medication adherence is ultimately the responsibility of the patient and they will only address it, when patients ask about it themselves (quote 4.4.4 in Table 7). GPs stressed the need for HCPs to shift mindset in this regard, as inquiring patients’ about satisfaction with their medication is not a moment of judgement, but rather a moment to assess the needs of a specific patient regarding their medication (quote 4.4.5 in Table 7).

##### High drop-out rates among HCPs

One GP and her NP dropped out of the project due to the high time investment needed for a small number of patients. Additionally, four other NPs dropped out or were temporarily unavailable due to long-term illness, maternity leave, or quitting their job during the intervention period.

## Discussion

### Main findings

In the present study, the previously developed tailored medication adherence intervention ‘Support for diabetes’ was digitalized and a protocol for collaboration between HCPs was developed based on input from patients and HCPs. However, implementation of the intervention in its improved digitalized form appeared not feasible. Three main reasons were derived from HCPs perspective. First, GP and CP ICT systems lack features to properly identify non-adherent and sub-optimally controlled T2DM patients. Second, NPs were the central actors in this intervention, but had difficulties discussing delicate topics such as medication non-adherence with T2DM patients. Third, a well-established collaboration between CP and GP teams appeared an important prerequisite for implementing this intervention, but this collaboration was suboptimal in one of the participating healthcare centers.

### Comparison with findings from other studies

Our findings partly align with earlier findings in other pilot and feasibility studies aimed at improving adherence. Refill adherence is generally considered a reliable and objective measure of medication adherence[24], but limitations when used as eligibility criterion have been reported. For example, when a patient’s dose is reduced or halted without a new prescription being written, patients seemed non-adherent to medication based on refill adherence, while in reality they are not [25]. Our study adds to these findings by showing that the repeat dispensing service of pharmacies also decreases the reliability of refill adherence to identify patients that are non-adherent. This is because the repeat dispensing service automatically generates a new prescription after a given time period of which the patient receives an alert to come and collect their medication. This caused issues because the refill adherence system would not be aware that a patient did not pick up their previous batch of medication when a new one was automatically made.

The current ‘Support for diabetes’ intervention was based on an earlier intervention by Adhien et al. The main limitations reported in the first ‘Support for diabetes’ intervention by Adhien et al. [15] were a lack of collaboration between GPs and CPs and the intervention being too time-consuming. Unlike previously suggested [19], digitalization of the intervention did not lead to a decrease in time spent on the intervention by HCPs. This can largely be explained by shortcomings in the ICT for the selection of eligible patients. The A1c values of non-adherent patients had to be checked manually by the NP and the GP, which was considered very time-consuming by the involved HCPs.

Another comparable intervention, The CATI intervention for hypertensive patients [26, 27] concluded that the selection of non-adherent patients should be based on both refill adherence and relevant biometric values. This was applied in this study, but this addition proved challenging in new ways. Medication adherence based on pharmacy refill data was considered unreliable by GPs and NPs, mainly attributed to the repeat dispensing service. Improvements in ICT are needed to automatically update patients’ refill adherence data when medication is not picked up. Alternatively, future studies could also consider adding a supportive instrument designed to quickly identify non-adherent patients in a routine care setting. Examples would be the triage question set or the Pringle questions [28, 29].

Suboptimal collaboration is a common barrier for successful implementation of complex interventions in previous studies [15, 26, 27], also confirmed in this study. The collaboration protocol established in this project was aimed at minimizing existing differences in collaboration between different settings. This was done by providing each healthcare center with a clear description of tasks and responsibilities, rather than relying on existing distribution of labor. However, this did not lead to an improvement of collaboration between the HCP in the healthcare center where the collaboration was subpar, and was considered unnecessary by the HCP in the healthcare center in which the collaboration was already excellent prior to the intervention. This implies that a well-established collaboration is an important prerequisite for complicated medication adherence interventions.

Another challenge was the difficulties NPs experienced when discussing medication non-adherence with T2DM patients. This resulted in NPs not asking follow-up questions or canceling consults. This is in line with previous findings about NPs having difficulties addressing lifestyle behaviors in the Dutch primary care, where NPs often did not follow guidelines to address physical activity during consults with T2DM patients [30]. These findings can be described as a low implementation fidelity, i.e., the extent to which an intervention was delivered according to the protocol [31]. According to implementation fidelity theory [31], low fidelity can be explained by moderating factors such as participant responsiveness, intervention complexity, and facilitation strategies used. Participant responsiveness, or the way in which the intervention is perceived by the users, was generally negative. Especially GPs indicated that the time invested per patient was not in line with profit in health. This resulted in GPs not scheduling time off in NPs agendas to work on the intervention or not inquiring about the intervention. The given complexity of this tailored, multi-faceted intervention also makes it more vulnerable to differences in implementation [32], which was observed by a large difference in implementation between the two healthcare centers. Lastly, additional facilitation strategies used for implementation might have been useful. More specifically, NPs might have benefitted from a training more aimed towards motivational interviewing, as this has been shown to positively influence adherence [33].

### Strengths and limitations

A strength of the current study is the extensive context analysis and tailoring of a promising intervention based on wishes of involved HCPs. Furthermore, patients preferences were taken into account during the development of the intervention, which might have a positive effect on both patient retention and engagement in the study [34]. Additionally, the implementation process in this project was investigated thoroughly which resulted in numerous suggestions for improvement for future intervention studies. This was done by making use of rigorous qualitative research. For example, this study used investigator triangulation to enhance the credibility of the findings [35]. A limitation of this study is the inability to take all input from phase 1 (context analysis) into account during the implementation phase. More specifically, HCPs voiced their concerns regarding the eligibility criteria based on refill adherence due to the existence of the repeat dispensing service by pharmacies. Due to limitations in ICT systems, we were not able to control for the effects of repeat dispensing on refill adherence.

Another limitation is the transferability of the results found in the qualitative research in phase 1 and phase 4. In this feasibility study in one health care center participated in phase 1 and in phase 4, a second healthcare center. In addition, in these healthcare centers, pharmacists and general practitioners are in close proximity, which might already positively influence their collaboration. The results can therefore not be easily generalized to other healthcare settings. However, in this stage, the feasibility of the intervention rather than the transferability of the results was the main concern*.*Since this study involved only two focus groups with different participants in phase 1 and one focus group in phase 4, it is currently unknown if data saturation has been reached. Additionally, the NPs interviewed in this study might not have been fully open and honest due to their supervisor being present during the focus groups.

## Conclusions

The ‘Support for Diabetes’ intervention was improved and digitalized, but implementation in the Dutch primary appeared not feasible as of yet. Main reasons were the lack of sufficient ICT applications to monitor and select non-adherent patients, the lack of collaboration between HCPs, and difficulties experienced by NPs in discussing medication non-adherence with T2DM patients. Implementation of the ‘Support for diabetes’ intervention in its current form for the population selected in this study is therefore not recommended. Future studies should explore ways to support NPs in addressing medication non-adherence with their patients, e.g., by training their communication skills or by adding more support from another HCP, e.g., the community pharmacist in carrying out the intervention. As an increasing amount of care is shifting towards primary care, better alignment of GP and CP ICT systems and an improved collaboration between HCPs are important prerequisites to successfully implement complicated tailored interventions as aiming to improve care to support medication adherence.

## Supplementary Information

**Additional file 1**: Appendix A. Topic lists Topic list for focus group with T2DM patients (N = 11) to gain insight into current standards of care to support medication adherence (phase 1 – context analysis). Appendix BA. Topic listsTopic list for focus group with HCPs (N = 5) to gain insight into current standards of care to support medication adherence (continued) Topic list for focus group with HCPs (phase 1 – context analysis). Appendix C. Topic list for focus group with HCPs (n = 6) and interviews (n=4) to gain insights into the implementation of the improved and digitalized ‘Support for Diabetes’ intervention using components of the RE-AIM model (phase 4 – testing the implementation). Appendix DB. Collaboration protocol for HCPs for the Support for Diabetes project. Appendix EC. Screenshot of digitalized intervention ‘Support for Diabetes’ with example of shared function, in which answers by one HCP are visible for all involved HCPs (see arrow). Appendix F. Schematic overview of the improved and digitalized ‘Support for Diabetes’ intervention

## Data Availability

Not applicable.
