# Supplementary material for: Feasibility study of a digitalized nurse practitioner-led intervention to improve medication adherence in type 2 diabetes patients in Dutch primary care
Source: Pilot Feasibility Stud. 2021 Aug 7;7:152. doi: 10.1186/s40814-021-00892-2 (PMC8349070; doi:10.1186/s40814-021-00892-2)
Supplement: Supplementary file 1 — Additional file 1: Appendix A. Topic lists Topic list for focus group with T2DM patients (N = 11) to gain insight into current standards of care to support medication adherence (phase 1 – context analysis). Appendix BA. Topic listsTopic list for focus group with HCPs (N = 5) to gain insight into current standards of care to support medication adherence (continued) Topic list for focus group with HCPs (phase 1 – context analysis). Appendix C. Topic list for focus group with HCPs (n = 6) and interviews (n=4) to gain insights into the implementation of the improved and digitalized ‘Support for Diabetes’ intervention using components of the RE-AIM model (phase 4 – testing the implementation). Appendix DB. Collaboration protocol for HCPs for the Support for Diabetes project. Appendix EC. Screenshot of digitalized intervention ‘Support for Diabetes’ with example of shared function, in which answers by one HCP are visible for all involved HCPs (see arrow). Appendix F. Schematic overview of the improved and digitalized ‘Support for Diabetes’ intervention [file 40814_2021_892_MOESM1_ESM.docx]

**Appendix A; Topic list for focus group with T2DM patients (N = 11) to gain insight into current standards of care to support medication adherence** *(phase 1 – context analysis)*

| **A Medication adherence** |
| --- |
| A1. Do you believe all medication that is prescribed to you, is actually beneficial to you?  A2. What are factors that might influence whether you take the medication or not? |
| **B Medication support and information provided** |
| B1. What do you think about the quality of the information provided about your medication by the NP/GP? And what about the pharmacy?  B2. If there are decisions made about your medication regimen, how are these decisions made? Does this differ between HCPs?  B3. Have you ever had a consult with the CP concerning the medication that you take? If yes, who initiated this consult? What was your general perception of this consult? |
| **C Multidisciplinary care and collaboration** |
| C1. In a previous project, we noticed a suboptimal collaboration between HCPs. Is this something you as a patient recognize as well?  C2. Which of your HCPs is best aware of the medication that you take?  C3. Who, according to you, should have the final say in your medication regimen?  C4. Currently, a CP has limited authorizations when it comes to making adjustments to medication. Do you think this should change?  C5. Do you use medication for different conditions? If so, to what extent does the GP/NP take this into account? And the CP?  C6. (whiteboard/brainstorm question)  What do you think is important in a good collaboration between HCPs? What addition or improvement would you like to see most? |
| **D Final question** (Ask each individual patient) |
| D1. Is there anything else that we did not talk about, which you would like to mention? |

**Appendix B; Topic list for focus group with HCPs (N = 5) to gain insight into current standards of care to support medication adherence**  *(phase 1 – context analysis)*

| 1. **Medication adherence** |
| --- |
| A1. What does good medication support look like in your eyes?  A2. What are factors that might influence good medication support?  A3. What influences could the mentioned barriers for sufficient medication support have on the medication adherence of T2DM patients? |
| 1. **Collaboration, communication and the role of digitalization** |
| **Key questions**  B1. Based on experience from the previous version of this intervention, a suboptimal collaboration was an important barrier for good medication support for T2DM patients. Is that recognizable? Why (not)?  B2a. What are factors that influence proper collaboration for medication support for T2DM patients? Welke factoren stimuleren een goede samenwerking in de diabetes medicatiezorg?  B2b. Which factors can hamper an effective collaboration in adherence supportive care?  *You are all users of Pharmacom/Medicom ICT systems.*  B2. What role does Pharmacom/Medicom play in the communication and collaboration?  **Prime questions (optional, ask solely if response on above questions is lacking)**  B1. In which situations would you ask support or advice from a co-worker from a different practice (e.g. the GP or CP)? *Over Pharmacom/Medicom*  B2. Which parts of Pharmacom/Medicom are essential for a good collaboration and communication? And to what extent are these features currently present?  B3. What other means of communication or collaboration do you have, and to what extent are they used? |
| 1. **Division of labour** |
| **Key questions**  C1. Which HCP has end responsibility over the medication adherence of T2DM patients?    C2. *Involes small exercise. First all HCPs answer the questions below individually on a piece of paper, afterwards the answers are discussed in the group and differe*nces are explored. Each question has four answer options (GP, CP, CP assistant or NP).   C2a. Who should take the initiative in signaling non-adherent T2DM patients?  C2b. Who should approach non-adherent T2DM patients?  C2c. Who should be responsible for treating non-adherent T2DM patients?  **Prime questions (optional, ask solely if response on above questions is lacking)**  C1. Which work agreements are essential for good medication supportive care for T2DM patients?  C2. Who is primarily responsible for providing the proper information about medication to T2DM patients? |
| 1. **Final question** (Ask each individual HCP) |
| D1. Is there anything else that we did not talk about, which you would like to mention? |

**Appendix C. Topic list for focus group with HCPs (n = 6) and interviews (n=4) to gain insights into the implementation of the improved and digitalized ‘Support for Diabetes’ intervention using components of the RE-AIM model** *(phase 4 – testing the implementation)*

| **A Introduction** |
| --- |
| A1. What was the most important reason to participate in this project? |
| **B Reach** |
| *Key questions*  B1. We have experienced difficulties in selecting eligible patients. What is the cause according to you?  B2. What did patients that did participate generally think of the project?  *Prime questions*  B3. Follow-up question for 1: Were the selected patients suitable for this intervention ?  follow-up question for 3: What did you think of the patients insight in their own medication adherence? Were the barriers that patients would be divided into recognizable for patients and helpful? |
| **C Implementation** |
| *Key questions*  C1. What were the biggest challenges for you in this project?  C2. How did you generally experience the collaboration between CP and GP staff members? (follow-up: how could this have been better?)  C3. What influence did the digitalization of the intervention have?  C4. What would you do differently, if you would do this project again?  *Prime questions*  C5. The CP had only a small role in this project. Should this role be increased? If so, how?  C6. Did the collaboration protocol help during this intervention? How often did you use it ? |
| **D Adoption** |
| *Key questions*  D1. How did you get involved in this project? how did that process go?  D2. What was your general perception of the training given?  D3. How well did the training prepare you for the tasks in this project?  *Prime questions*  D4. Ask about the details of the training; duration, missing or unneccesary topics, other remarks  D5. What kind of knowledge do you have right now, which you would like to have had earlier in this project? |
| **E Effectiveness** |
| *Key questions*  E1. How did you experience the consults with patients?  E2. To what extent does this intervention differ from usual care?  E3. To what extent do you think this intervention helped T2DM patients maintain a better A1c/medication adherence?  *Prime questions*  E4. What are important succesful aspects of the intervention? And which aspects are unnecessary?  E5. What did you think about the quick barrier scan and the barrier profiles?  E6. Wat did you think about the five modules that were tailored tot he individuel barriers of patients? |
| **F Maintenance** |
| *Key questions*  F1. Is the intervention applicable in the usual care in its current form? If no, what changes are neccesary?  F2. Are there things that you expected us (the project team) to supply you or help you with, which we did not do?  F3. Zijn are there any final remarks you would like to add?  *Prime questions*  F4. Which advice would you give to the organization which provided us with the funding to carry out this project? |

**Appendix D; collaboration protocol for HCPs for the Support for Diabetes project**

Step 1a. Selection of patients

Step 1b. Signaling and plan for action

Step 2. Invite patients and baseline measurements

Step 3. Conducting the Support for Diabetes intervention

Step 4. Follow-up

**Step 1a. Selection of patients**

*What?* Selecting non-adherent T2DM patients (based on refill adherence CP or during patient consults)

*Who*? Pharmacist (electronic search), everyone (during consults)

*When*? CP delivers a list of non-adherent T2DM patients quarterly to each GP. When non-adherence is observed in between these timeframes, communicate with the treating NP

*How*? Ask open questions, be alert during consults. Use the ‘action list’ in Pharmacom/Medicom for communication

**Step 1b. Signaling and plan for action**

*What?* Inclusion of DM2 patients (non-adherent + sub-optimally controlled)

*Who?* GP (optionally with input from treating NP).

*When?* The GP does this quarterly after receiving the list of non-adherent T2DM patients from the CP

*How?* Based on the list provided by the CP, check whether listed T2DM patients are also sub-optimally controlled based on current guidelines. If yes, let the NP know to invite the patient for the intervention.

**Step 2. Invite patients and baseline measurements**

*What?* Invite the selected patients by means of telephone. Send the letter of participation and questionnaires by mail.

*Who?* NP or GP assistant.

*When?* By request of the GP.

*How?* Within a month of receiving the list with non-adherent T2DM patients from the CP, check corresponding A1c levels and invite eligible T2DM patients.

**Step 3. Conducting the ‘Support for Diabetes’ intervention***What?* Conduct the intervention ‘Support for Diabetes’ with the selected patients.

*Who?* NP.

*How?* Based on the protocol ‘Support for Diabetes’ (see screenshot Appendix C for an example)

*When?* Within one month after receiving the list of non-adherent T2DM patients from the CP. Remember to check the follow-up time of 3 months.

**Step 4. Follow-up**

*What*? Send questionnaire medication adherence (ASK-12 and BMQ)

*Who*? Assistant or NP

*When*? 3 months after conducting the intervention ‘Support for Diabetes’

How? Send the questionnaire by mail, which can be found in the project map and in your inbox.

**Appendix E; Screenshot of digitalized intervention ‘Support for Diabetes’ with example of shared function, in which answers by one HCP are visible for all involved HCPs (see arrow).**

**
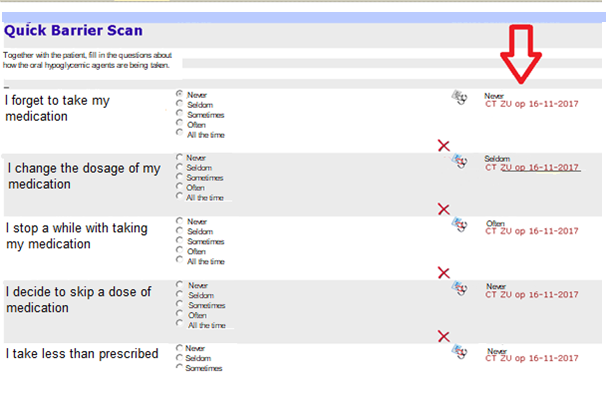
**

Appendix F: Schematic overview of the improved and digitalized ‘Support for Diabetes’ intervention
